# Neuroprotective Mechanisms of Ginsenoside Rb1 in Central Nervous System Diseases

**DOI:** 10.3389/fphar.2022.914352

**Published:** 2022-06-02

**Authors:** Liang Gong, Jiayi Yin, Yu Zhang, Ren Huang, Yuxuan Lou, Haojie Jiang, Liyan Sun, Jinjing Jia, Xiansi Zeng

**Affiliations:** ^1^ Jiaxing University Medical College, Jiaxing, China; ^2^ Department of Clinical Medicine, Jiaxing University Medical College, Jiaxing, China; ^3^ Research Center of Neuroscience, Jiaxing University Medical College, Jiaxing, China

**Keywords:** central nervous system diseases, ginsenoside Rb1, neuroprotection, mechanisms, antioxidant

## Abstract

Panax ginseng and Panax notoginseng, two well-known herbs with enormous medical value in Asian countries, have a long usage history in China for the therapy of some diseases, such as stroke. Ginsenoside Rb1 is one of most important active ingredients in Panax ginseng and Panax notoginseng. In the last two decades, more attention has focused on ginsenoside Rb1 as an antioxidative, anti-apoptotic and anti-inflammatory agent that can protect the nervous system. In the review, we summarize the neuroprotective roles of ginsenoside Rb1 and its potential mechanisms in central nervous system diseases (CNSDs), including neurodegenerative diseases, cerebral ischemia injury, depression and spinal cord injury. In conclusion, ginsenoside Rb1 has a potential neuroprotection due to its inhibition of oxidative stress, apoptosis, neuroinflammation and autophagy in CNSDs and may be a promising candidate agent for clinical therapy of CNSDs in the future.

## Introduction

Panax ginseng and Panax notoginseng are two valuable medicinal herbs in the genus Panax, family Araliaceae (Wang et al., 2016). The curative effects of Panax ginseng and Panax notoginseng were first detailedly recorded by Shizhen Li in “Compendium of Materia Medica,” which was praised by Darwin as “the Encyclopedia of ancient China.” Panax ginseng and Panax notoginseng have a wide and significant application in medicinal purposes and economic values (Qiao et al., 2018). In China Panax ginseng has a more than 5,000 years of application history (Yun, 2001) and was recorded in the world’s oldest pharmacopeia of medicinal herbs and plants, “Shennong’s Herbal Classic” (Shi et al., 2019). Panax ginseng mainly grows in the mountains of East Asia countries, particularly in China, Korea and Japan (Baeg and So, 2013). Shizhen Li described Panax ginseng as a magic medicine that can almost cure all diseases. Since the content of active ingredient in Panax notoginseng is higher than that in Panax ginseng, Panax notoginseng enjoys a reputation for “The King in Panax.” In modem times, they have received considerable interest due to their extensive application in healthcare products, clinical therapy, and as foods and food additives in the whole world (Yang et al., 2014) because they could relieve stress and fatigue, prevent aging, increase vigor and strengthen the body and mind (Choi, 2008; Kim et al., 2015). Panax ginseng and Panax notoginseng are often used to slow down the symptoms, such as traumatic injury, blood stasis, swelling and pain (Tianhong et al., 2014; Chen et al., 2017; Wu et al., 2018). The ginsenosides, chemical constituents found in Panax ginseng and Panax notoginseng, can inhibit the effects of inflammatory cytokines, block signaling pathways that induce inflammation, and inhibit cells that participate in inflammatory processes (de Oliveira Zanuso et al., 2022). What’s more, increasing evidence has demonstrated that ginsenosides are involved in neuroprotective effects in the central nervous system diseases due to their antioxidant, anti-apoptotic, and anti-inflammatory features (Lu et al., 2022).

## Ginsenoside Rb1

**FIGURE 1:**
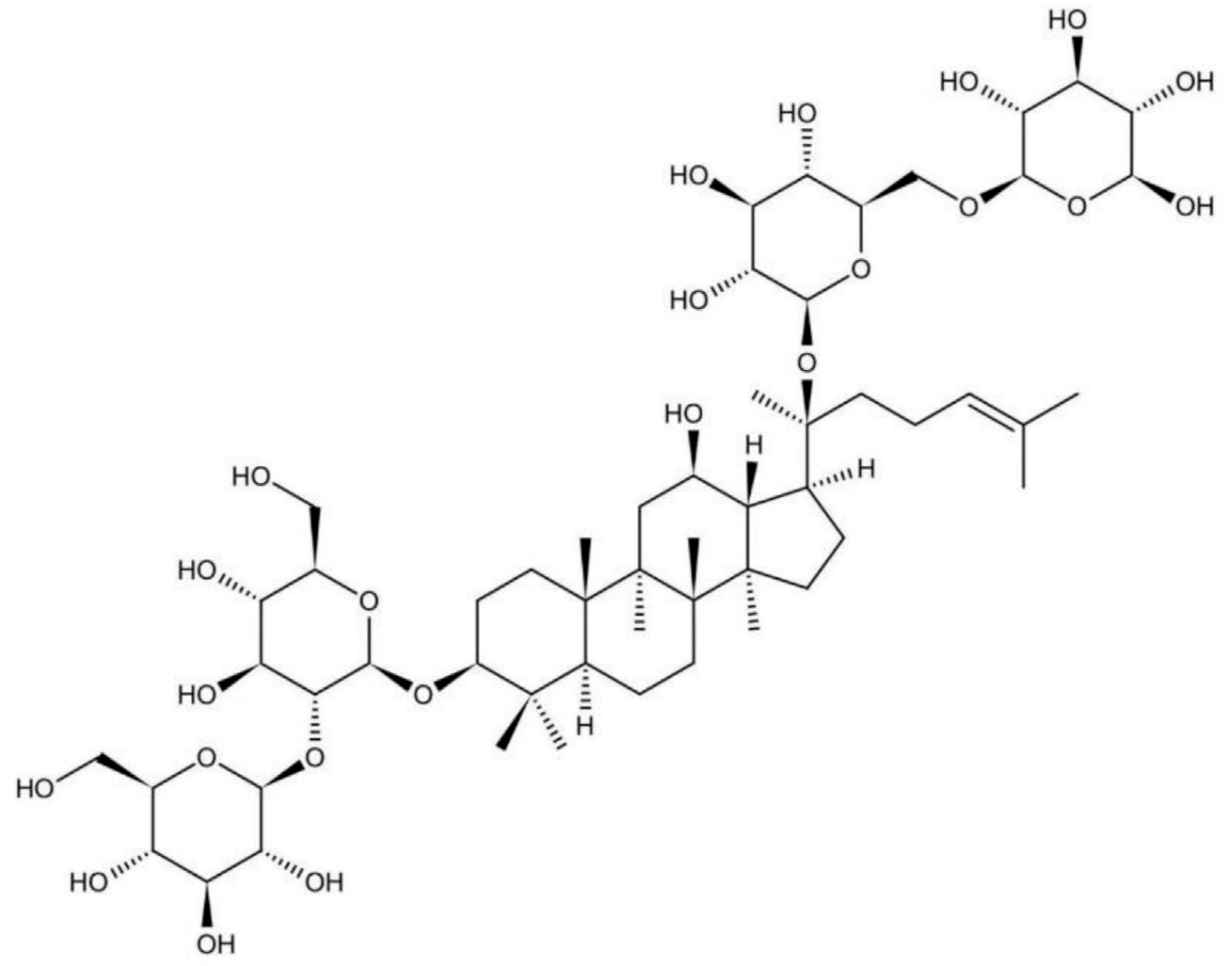
The structure of ginsenoside Rb1.

Many studies support that the beneficial effects of Panax ginseng attributed to ginsenosides (Qi et al., 2011). Triterpenoid plays an important role in the medical value of Panax ginseng and Panax notoginseng (Ng, 2006). Triterpenoid is constituted mainly by ginsenoside Rb1, ginsenoside Rb2, and notoginsenoside R1, among which ginsenoside Rb1 takes up a tremendous part. Ginsenoside is a kind of steroids, also known as triterpenoid saponins. All the ginsenosides have the similarity in the basic structure, containing sterane steroid nuclei arranged into four rings by 30 carbon atoms (Cheng et al., 2019). Results also indicate that the function of ginsenoside Rb1 is superior to the function of ginsenoside Rb2, because ginsenoside Rb1 is a panaxtriol with two sugars and ginsenoside Rb2 is a panaxtriol with four sugars (Kim et al., 2014). The structure of ginsenoside Rb1 is shown in Figure 1.

## Pharmacological Effects of Ginsenoside Rb1

With the development of the study, ginsenoside Rb1 presents antioxidant, anti-apoptotic, and anti-inflammatory properties. In a cell free system, ginsenoside Rb1 can significantly and selectively scavenge hydroxyl radical and hypochlorous acid, two of the strongest reactive oxygen species (ROS), and protect biomacromolecules from oxidative damage (Lu et al., 2012). Ginsenoside Rb1 could inhibit mitochondria-, endoplasmic reticulum stress- and death receptor-mediated apoptotic pathways (Ke et al., 2021; Shaukat et al., 2021). Our previous study demonstrated that ginsenoside Rb1 inhibited oxidative stress-induced endoplasmic reticulum stress in rat PC12 cells (Zeng et al., 2015). What’s more, treatment with ginsenoside Rb1 attenuated tumor necrosis factor-α (TNF-α)-induced inflammation by inhibiting the activation of c-Jun N-terminal kinase (JNK) and p38 pathways in human umbilical vein endothelial cells and further suppressed the nuclear factor-kappa B (NF-κB) signaling and downregulated the expression of inflammatory factors (Zhou et al., 2017). In addition, ginsenoside Rb1 has the ability to regulate autophagy (Liu et al., 2020). It’s believed that oxidative stress, apoptosis, inflammation and autophagic dysfunction contribute to a variety of diseases, especially the central nervous system diseases (CNSDs). CNSDs, including neurodegenerative diseases, cerebral ischemia injury, depression and spinal cord injury, which are always difficult to cure clinically. Therapeutic drugs used clinically fail to block the development of diseases or are proved to produce severe side effects. Thus, there is an urgent need to develop new drugs to treat these diseases. In the last two decades, ginsenoside Rb1 is reported to play potent neuroprotection in rodent models of CNSDs. In this review, we summarize the neuroprotective roles of ginsenoside Rb1 and highlight its potential molecular mechanisms.

## Ginsenoside Rb1 in Neurodegenerative Diseases

### Alzheimer’s Disease

Alzheimer’s disease (AD) is the most common neurodegenerative disease, characterized by progressive cognitive and behavioral impairment (Mao et al., 2018). The pathological features of AD are Amyloid β (Aβ) deposition (Harrison et al., 2021), tau protein hyperphosphorylation (Xia et al., 2021) and loss of hippocampal neurons (Edler et al., 2017).

Some *in vitro* studies showed that ginsenoside Rb1 protected against Aβ-induced cytotoxicity in various cells (Qian et al., 2009; Xie et al., 2010). Changhong and others found that ginsenoside Rb1 treatment could serve as an activator of peroxisom proliferator-activated receptor-γ (PPARγ) and reduce the level of cholesterol and further lowered the cytotoxicity of Aβ_25-35_ in PC12 cells (Changhong et al., 2021). Several *in vivo* studies revealed the neuroprotective roles and potential mechanisms in various AD models. Oral administration of ginsenoside Rb1 significantly shortened the escape latency in the Morris water maze (MWM) test and reduced the number of errors in the passive avoidance task by decreasing protein expression levels of ASC, caspase-1 and Aβ, repairing neuronal cells loss and inhibiting the activation of astrocyte and microglia in hippocampus of SAMP8 mice (Yang et al., 2020c). More importantly, ginsenoside Rb1 was more effective than another ginsenoside, Rg1, in SAMP8 mice. One interesting study showed that ginsenoside Rb1 given orally is completely metabolized to 20-O-beta-D-glucopyranosyl-20(S)-protopanaxadiol (M1) to exert neuroprotective role (Tohda et al., 2004). Ginsenoside Rb1 improved memory and cognitive ability of streptozotocin (STZ)- injected mice and also relieved glucose intolerance induced by STZ injection by enhancing insulin sensitivity (Yang et al., 2020b). These beneficial effects of ginsenoside Rb1 is most likely mediated by upregulating the expression of NMDAR1 and IDE in the hippocampus through inhibiting the activity of CDK5/p35. Ginsenoside Rb1 treatment decreased the levels of Bax and cleaved caspase-3, upregulated the level of Bcl-2 in the hippocampus (Wang et al., 2018c), suggesting that ginsenoside Rb1 inhibited Aβ_1-40_-induced mitochondrial apoptosis pathway.

The abnormal deposition of Aβ, a common induction pathway in AD (Hardy and Selkoe, 2002), exhibits neurotoxicity and may lead to a complex array of responses, including an inflammatory cascade (Streit, 2004). AD inflammation and thrombosis are promoted by Aβ through interaction with circulating protein XII and fibrinogen (Zamolodchikov and Strickland, 2016). Pro-inflammatory cytokines such as interleukin-1β (IL-1β), IL-6, IL-10, and TNF-β were found to have increased expression in the brain and cerebrospinal fluid of AD patients (Mrak and Griffin, 2005; Jiang et al., 2011). Interestingly, ginsenoside Rb1 mitigated the isoflurane/surgery-induced cognitive impairment- and synapse dysfunction *via* decreasing levels of ROS, TNF-α and IL-6 in the mice hippocampus, suggesting that the mechanisms refer to inhibiting oxidative stress and neuroinflammation (Miao et al., 2017). Glial fibrillary acidic protein (GFAP), an astrocyte marker, is associated with memory impairment and neuronal reduction (Murphy et al., 2003). In a Aβ_1-40_-induced AD model, the expression of inflammation-related genes Aβ, IL-1β, and GFAP were decreased in the hippocampus of rats after ginsenoside Rb1 injection (Lin et al., 2019), indicating that ginsenoside Rb1 can reduce the neuroinflammation in AD. The increased number of hippocampal neurons in CA1 area may be involved with the ability of ginsenoside Rb1 promoting the proliferation and differentiation of neural stem cells (NSC) in AD model (Zhao et al., 2018b). (Table 1).

**TABLE 1:** The neuroprotection of ginsenoside Rb1 in AD.

AD models	Dose of Rb1	Effects	Mechanisms	References
Aβ25-35-treated PC12 cells	50 μM	Inhibiting cytotoxicity of Aβ	Antioxidant	Changhong et al. (2021)
SAMP8 mice	60 μmol/kg orally for 8 weeks	Improving memory and cognitive ability	Anti-neuroinflammation, inactivating astrocyte and microglia	Yujie Yang et al. (2020c)
STZ-injected mice	30 mg/kg	Improving memory and cognitive ability	Upregulating the expression of NMDAR1 and IDE in the hippocampus	Ranyao Yang et al. (2020b)
Aβ1-40-treated rats	25 mg/kg for 14 consecutive days	Preventing cognitive deficit	Anti-apoptosis in the hippocampus	Yiru Wang et al. (2018c)
Aβ1-40-treated rats	25 mg/kg for consecutive 2 weeks	Improving learning and memory ability	Anti-neuroinflammation	Lin et al. (2019)
Hippocampal injection of Aβ1-40	10 mg/kg daily for 30 days	Promoting proliferation and differentiation of NSCs	N/A	Jiwei Zhao et al. (2018b)
Okadaic acid-treated brain slice	240 μM for 4 h	N/A	Suppressing tau hyperphosphorylation and upregulating BDNF expression	Wang et al. (2013)
Aβ25-35-treated rat cortical neurons	40 μM for 24 h	Inhibiting cytotoxicity of Aβ	Attenuating tau hyperphosphorylation and regulating Ca^2+^ signaling	Chen et al. (2008)

Neurofibrillary tangles (NFTs), composed of hyperphosphorylated microtubule-associated protein tau, are a defining pathological feature of AD. It is hypothesized that hyperphosphorylation of tau impairs the microtubule-stabilizing function of tau, leading to the formation of paired helical filaments and neuronal death (Liu et al., 2003). In spite of the fact that Aβ aggregation is considered an important causative factor of AD, there are great correlations between clinical symptoms, atrophy and brain damage and the appearance of tau aggregation (Bennett et al., 2017). Ginsenoside Rb1 can reduce the expression of phosphorylated tau protein in brain slices of rat model of AD and effectively reduce the formation of NFTs (Wang et al., 2013). Overexpression of glycogen synthase kinase-3β (GSK3β), a tau protein kinase, induced hyperphosphorylation of tau protein in cellular and animal models (Lucas et al., 2001). Brain-derived neurotrophic factor (BDNF) inhibited tau protein phosphorylation by suppressing the activity of GSK3β (Liu et al., 2003). Ginsenoside Rb1 inhibits tau protein phosphorylation by upregulating BDNF and therefore has a preventive effect in AD (Wang et al., 2013). Increasing studies have shown that Aβ induced the hyperphosphorylation of tau. Ginsenoside Rb1 inhibited fibrillar Aβ_25-35_-induced tau hyperphosphorylation in primary cultured cortical neurons *via* inactivating Ca^2+^/calpain/CDK5 signal pathway (Chen et al., 2008) (Table 1).

Several omics researches revealed the neuroprotection of ginsenoside Rb1 in AD. An RNA sequencing study demonstrated ginsenoside Rb1 could regulate the expression of genes related to nervous system development and mitogen-activated protein kinase (MAPK) signaling pathway in SAMP8 mice (Zhang et al., 2017). Ginsenoside Rb1 treatment also showed a potential to upregulate the expression of proteins, such as CAP1, CAPZB, TOMM40, and DATN which are essential for growth cone morphology and neurite outgrowth according to results of a proteomics study (Hwang et al., 2016). In addition, a metabolomic study revealed that ginsenoside Rb1 displayed anti-AD effects through regulating lecithin and amino acid metabolism (Li et al., 2015).

These researches demonstrated that ginsenoside Rb1 have potent effects in alleviating the pathological features of AD, suggesting ginsenoside Rb1 may be a competitive candidate for AD therapy.

### Parkinson Disease

Parkinson Disease (PD), one of the most common neurodegenerative diseases (Obeso et al., 2017), is characterized by the loss of dopaminergic neurons in the substantia nigra of midbrain (Zeng et al., 2014a). Actually, increasing studies have showed that both genetic and environmental factors may lead to PD inspite of the unknown pathology of PD (Kim and Alcalay, 2017). Based on current studies, the mutations of several genes, including *α-synuclein*, *LRRK2*, *PINK1*, *Parkin*, *DJ-1*, *VPS35*, and *GBA1*, lead to the onset of PD (Zeng et al., 2018c). Various biological processes, such as dopamine metabolism, mitochondrial dysfunction, endoplasmic reticulum stress, impaired autophagy, and deregulation of immunity could lead to the loss of dopaminergic neurons (Zeng et al., 2018c).

Normally, pathologic changes of PD contain the appearance of Lewy bodies and the mass death of dopaminergic neurons (Simon et al., 2020). The misfolded of α-synuclein (α-syn) is a classic sign in PD. Study shows that the initial α-syn aggregations is neurotoxic since they cause the death of the cells (Mehra et al., 2019). Ardah et al. (2015) found that ginsenoside Rb1 could inhibit the toxicity and aggregation of α-syn; What’s more, ginsenoside Rb1 exhibited a strong ability to decompose preformed fibrils. Mechanistically, ginsenoside Rb1 bonds to soluble non-toxic oligomers with no β-sheet content, making it susceptible to proteinase K digestion. Ginsenoside Rb1 attenuated the lipopolysaccharide (LPS)-induced depletion of dopamine and its metabolites in the striatum, inhibiting dopamine (DA)rgic neuron degeneration in the substantial nigra *via* inhibiting the activation of microglia in substantial nigra by downregulating LPS-induced activation of NF-κB signaling pathway (Li et al., 2019a). An earlier study showed that ginsenoside Rb1 significantly increased the numbers and lengths of neurites of surviving DA neurons although it could not prevent cell death by glutamate challenge (Radad et al., 2004). 1-methyl-4-phenyl-1,2,3,6-tetrahydropyridine (MPTP) is a commonly used reagent to build PD model in mice (Zeng et al., 2018b), which could cross the blood-brain barrier (BBB, acting as the gatekeeper of the CNS in maintaining the delicate homeostasis of the brain), and be metabolized into the potent dopaminergic neurotoxin 1-methyl-4-phenylpyridinium ion (MPP^+^) by monoamine oxidase B in glial cells (Langston, 2017). It was reported for the first time that ginsenoside Rb1 treatment ameliorated motor deficits, prevented DA neuron death, and suppressed the expression of α-synuclein and astrogliosis in the MPTP mouse model of PD (Zhang et al., 2018). Glutamate plays a role in exciting neurons and promoting them to produce action potentials in CNS. *In vivo*, the appropriate level of glutamate is maintained by astrocytic glutamate transporters. However, too much endogenous glutamate may cause the death of the excitotoxic neurons, which may be connected with PD (Pajarillo et al., 2019). Zhang et al. (2018) found that ginsenoside Rb1 could suppress the excitotoxicity of glutamate by increasing glutamate transporter expression *via* nuclear translocation of NF-κB and modulates synaptic transmission in MPTP model of PD. In addition, ginsenoside Rb1 attenuated MPTP-induced cognitive impairment and dysfunctional gait dynamic *via* regulating gamma-aminobutyric acid (GABA)ergic transmission in the prefrontal cortex (PFC) (Liu et al., 2019). Administration of ginsenoside Rb1 also improved the memory deficiency of MPTP-treated mice *via* promoting hippocampal CA3 α-syn monomer expression, restoring the glutamate in the CA3-schaffer collateral-CA1 pathway, and sequentially increasing postsynaptic density-95 (PSD-95) expression (Qu et al., 2019) (Table 2). These researches suggest that ginsenoside Rb1 may serve as a potential therapeutic agent for PD.

**TABLE 2:** The neuroprotection of ginsenoside Rb1 in PD.

PD models	Dose of Rb1	Effects	Mechanisms	References
α-syn-treated BE(2)-M17 cells	5 μM	Restoring the decreased cell viability	Inhibiting α-syn fibrillation and toxicity	Ardah et al. (2015)
LPS-induced rat model	20 mg/kg for 14 consecutive days	Restoring DA and its metabolites in striatum and DA neurons degeneration in SN	Inactivating microglia in SN and inhibiting neuroinflammation	Da-Wei Li et al. (2019a)
MPTP-induced mouse model	40 mg/kg for 14 consecutive days	Ameliorating motor deficits, preventing DA neuron death	Attenuating glutamate excitotoxicity and modulating glutamatergic transmission pathways	Zhang et al. (2018)
MPTP-induced mouse model	10 mg/kg for 14 consecutive days	Mitigating MPTP-induced altered gait parameters and cognitive impairment	Regulating GABAergic transmission	Liu et al. (2019)
MPTP-induced mouse model	40 mg/kg for 14 consecutive days, starting 3 days before MPTP treatment	Improving memory deficiency	Improving synaptic plasticity	Qu et al. (2019)

### Other Neurodegenerative Diseases

A few studies also found that ginsenoside Rb1 showed neuroprotection in other neurodegenerative diseases, such as epilepsy and Huntington’s disease (HD). Ginsenoside Rb1 ameliorated pentylenetetrazol (PTZ)-injured longer seizure duration and shorter seizure latency, as well as the cognitive deficits and neuronal damage in rats *via* Rb1 dose-dependently increasing GSH levels, decreasing MDA levels, and activating Nrf2/ARE signaling (Shi et al., 2018). Its metabolic production, compound K, exerted anti-epileptic effects by promoting the hippocampal release of GABA and enhancing the GABA_A_ receptor-mediated inhibitory synaptic transmission (Zeng et al., 2018). Nanomolar concentrations of ginsenoside Rb1 effectively protected YAC128 medium spiny striatal neurons from YAC128 HD model mice against glutamate-induced apoptosis and Ca^2+^ responses (Wu et al., 2009). Notably, the protective effect of ginsenoside Rb1 is stronger than others ginsenosides.

## Ginsenoside Rb1 in Cerebral Ischemia

Ischemic stroke is one of the leading causes of adult disability and death all over the world. Cerebral ischemia, caused by a significant blockage in cerebral blood flow, results in various pathological events such as oxidative stress, neuroinflammation response, excitatory neurotransmitter release and energy failure, which eventually lead to neuronal apoptosis and brain tissue necrosis (Kamel and Iadecola, 2012; Khoshnam et al., 2017). What’s more, the recovery of blood supply after a certain period of brain tissue ischemia caused the secondary cerebral ischemia-reperfusion (I/R) injury. Currently, only a limited number of drugs commonly used in clinic are available for treating ischemic stroke and the therapeutic efficacy of these drugs is still limited (Cheng et al., 2019). Therefore, exploring and developing other potential strategies and agents for preventing and treating ischemic stroke are extremely and urgently needed.

Ginsenoside Rb1 could effectively reduce the levels of oxidative stress to protect against cerebral ischemia-induced neuronal injury. Our previous study demonstrated that ginsenoside Rb1 notably inhibited the increase in malondialdehyde (MDA) concentration and restored the expression of thioredoxin-1 (Trx-1) and copper-zinc superoxide dismutase (SOD-1) (Zeng et al., 2014b). As one of the two redox systems in cells, Trx plays crucial roles in maintaining the intracellular redox state. Trx-1 may serve as a therapeutic target in cerebral ischemia (Zeng et al., 2018a). Nrf2 is a primary antioxidant pathway and targets intracellular antioxidant genes (Carpenter et al., 2022). Ginsenoside Rb1 treatment could activate Keap1-Nrf2/ARE signaling pathway (Yang et al., 2020a). What’s more, ginsenoside Rb1 treatment at 24 h after surgery for 14 consecutive days activated cAMP/PKA/CREB signaling pathway in a distal middle cerebral artery occlusion (dMCAO) mouse model and promoted motor functional recovery and axonal regeneration (Gao et al., 2020), suggesting that ginsenoside Rb1 may serve as a potential clinically therapy drug during the recovery stage of stroke. Nrf2 and CREB are the transcription factor of Trx-1, which upregulate Trx-1 expression under stress (Im et al., 2012; Jia et al., 2016). Ginsenoside Rb1 treatment is able to improve the mitochondrial function in astrocytes in an oxygen-glucose deprivation/reoxygenation (OGD/R) model by inhibiting reactive oxygen species (ROS) formation and mitochondrial membrane potential (MMP) depolarization, increasing mtDNA content, activities of catalase (CAT), complexes I, II, III, and V and the level of ATP (Xu et al., 2019). (Table 3).

**TABLE 3:** The neuroprotection of ginsenoside Rb1 in cerebral ischemia.

Ischemia models	Dose of Rb1	Effects	Mechanisms	References
MCAO mice	40 mg/kg twice daily for 2 days prior to MCAO	Reducing infarction volume and alleviating neurological deficits	Antioxidant, pro-survival	Zeng et al. (2014b)
dMCAO mice	5 mg/ml e at 24 h after surgery for 14 consecutive days	Promoting motor functional recovery	Stimulating axonal regeneration and brain repair	Gao et al. (2020)
OGD/R in astrocytes	5 μM during OGD/R	Increasing the cell viability	Antioxidant	Xu et al. (2019)
MCAO rats	40 mg/kg after the onset of reperfusion	Promoting recoveries of neurological functions	Increasing nestin and BDNF; anti-apoptosis	Gao et al. (2010)
OGD/R in astrocytes	16.38 μg/ml for 72 h	N/A	Preventing the downregulation of AQP4, NGF and BDNF	Ya-Nan Li et al. (2019b)
MCAO mice	20–40 mg/kg after 3-h reperfusion for 2 days	Decreasing infarction, EB extravasation and brain edema, improving neurological deficits	Anti-neuroinflammation and antioxidant	Chen et al. (2015)
MCAO rats	12.5 mg/kg for 7 days before MCAO	Reducing infarction volume and alleviating the neurological deficit	Inactivating microglia in the penumbra and anti-neuroinflammation	Zhu et al. (2012)
MCAO rats	50–200 mg/kg	Improving the neurological deficits, decreasing infarct volume	Anti-apoptosis and anti-neuroinflammation	Anxin Liu et al. (2018a)
OGD/R in SH-SY5Y; two vessel occlusion	1–100 μM at 30 min prior to OGD for 24 h after recovery; 20–40 mg/kg at 15 min before ischemia	Inhibiting both OGD- and transient ischemia-induced neuronal death	Pro-survival and inhibiting autophagy	Luo et al. (2014)
MCAO mice	1–10 mg/kg after 3-h reperfusion	Decreasing infarction and brain edema, improving neurological deficits	Antioxidant and anti-neuroinflammation	Dong et al. (2017)

Administration of ginsenoside Rb1 could upregulate the expression of neurotrophic factor, which is beneficial for the neural survival. In a rat MCAO model, injection of ginsenoside Rb1 immediately after the onset of reperfusion significantly upregulated expression of BDNF, which induced the neurogenesis and promoted the recovery of neurological functions (Gao et al., 2010). In addition, Ginsenoside Rb1 also significantly increased the expression of nerve growth factor (NGF) and BDNF, as well as the expression of aquaporin 4 (AQP4), in OGD/R-induced rat astrocytes (Li et al., 2019b) (Table 3).

Increasing studies have suggested that neuroinflammation play an important role in the pathology of stroke. The main aim of acute stroke treatment is to rescue the ischemic penumbra or nonfunctional, yet still viable tissue surrounding the infarcted core. During ischemic stroke, the integrity of BBB tight junctions can be seriously destroyed due to blocked or reduced circulating blood flow (Meyers et al., 2011). Ginsenoside Rb1 protects loss of BBB integrity in ischemic stroke by suppressing neuroinflammation induction of matrix metalloproteinase-9 (MMP-9) and nicotinamide adenine dinucleotide phosphate oxidase 4 (NOX4)-derived free radical (Chen et al., 2015). A study reported that ginsenoside Rb1 administration markedly mitigated the activation of microglia in the ischemic penumbra by inhibiting the expression of TNF-α and IL-6 and the activation of NF-κB (Zhu et al., 2012), suggesting that ginsenoside Rb1 treatment is beneficial for salvaging the ischemic penumbra *via* inhibiting microglia-mediated neuroinflammation. High-mobility group box 1 (HMGB1) is released after focal cerebral I/R and aggravates brain tissue damage. Ginsenoside Rb1 treatment significantly inhibited the release of HMGB1 in MCAO rats, accompanied by decreasing the levels of NF-κB, TNFα, IL-6, and inducible nitric oxide synthase (iNOS) (Liu et al., 2018a), suggesting that the effects of ginsenoside Rb1 may be associated with the inhibition of HMGB1 inflammatory signals (Table 3).

The function of ginsenoside Rb1 in cerebral ischemia is related to common pro-survival pathways in cells. Ginsenoside Rb1 treatment upregulated the protein level of p-Akt at Ser473 and inhibited the elevation in protein levels of LC3II and Beclin1 and protected against OGD and transient global ischemia (two vessels occlusion)-induced injury (Luo et al., 2014). The recovery of brain damage after MCAO is significantly impaired in aged mice compared with young mice. Interestingly, long-term oral administration of ginsenoside Rb1 greatly prevented the injury in a dose-dependent manner by inhibiting oxidative stress and the activation of extracellular signal-regulated kinase 1/2 (ERK1/2) in aged mice (Dong et al., 2017). ERK1/2 activation is also a well-known pro-survival pathway, however, activation of mitogen-activated protein kinase/ERK may contribute to premature aging, and its inhibition has potential use in preventing cellular senescence as well as aging (Chappell et al., 2011) (Table 3).

These researches suggest that ginsenoside Rb1 may serve as a potential therapeutic agent for cerebral ischemia.

## Ginsenoside Rb1 in Depression

Depression, a common psychiatric disease and the leading cause of disability worldwide (Lopez and Murray, 1998), is a heterogenous diagnostic concept consisting of a set of different symptoms, with decreased mood, anhedonia, and reduced energy defined as core symptomatologies in ICD-10 and depressed mood and loss of interest and pleasure in DSM-5 (Hoflich et al., 2019). Depression is often associated with chronic illnesses (Evans et al., 2005) or other mood disorders such as co-morbid anxiety (Menard et al., 2016). At present, it is generally believed that dysfunction of monoamine neurotransmitters and their receptors, decrease in neurotrophic factors, neuroinflammation, activity enhancement of HPA axis are involved in the pathogenesis of depression. Monoamine oxidase inhibitors (MAOI), selective serotonin reuptake inhibitors (SSRI) or serotonin norepinephrine reuptake inhibitors (SNRI) are used clinically to treat moderate to severe depression (Walker, 2013). Unfortunately, these inhibitors are well recognized to produce a number of undesirable side effects that often lead to patient noncompliance. With the in-depth study of traditional Chinese medicine, people began to seek active ingredients from natural products for the treatment of depression. Increasing evidence demonstrated that ginsenoside Rb1 could significantly improve the depressive-like behaviors in various rodent models.

In a lipopolysaccharide (LPS)-induced depressive model, ginsenoside Rb1 significantly suppressed peripheral and hippocampal inflammation *via* MAPK/NF-κB signaling, improved impaired glucocorticoid receptor and inhibited activity of indoleamine 2,3-dioxygenase (Liang et al., 2022). The authors also found that ginsenoside Rb1 increased the levels of 5-HT and expression of 5-HT_1A_ receptor. The treatment with 5-HT_1A_ receptor antagonist, NAN190 or 5-HT_2A_ receptor antagonist, ritanserin, reversed the antidepressant-like effect of ginsenoside Rb1 in chronic unpredictable mild stress (CUMS) mice (Yamada et al., 2011; Wang et al., 2018a), suggesting that serotonergic receptor may be involved in the antidepressant-like effect of ginsenoside Rb1. Besides, dopaminergic and noradrenergic systems are also involved in the antidepressant-like effects of ginsenoside Rb1 (Wang et al., 2017). Rd, F2, compound K, Rh2, and Rg3 were identified as the metabolites of ginsenoside Rb1. Liang et al. (2022) found that F2 exerted antidepressant-like effects and it showed higher activity than ginsenoside Rb1 against depression (Table 4).

**TABLE 4:** The neuroprotection of ginsenoside Rb1 in depression.

Models	Dose of Rb1	Effects	Mechanisms	References
LPS-induced depressive mice	10–20 mg/kg daily for 11 consecutive days	Ameliorating LPS-induced Aepressive-like behaviors	Anti-inflammation, improving impaired Glucocorticoid receptor	Liang et al. (2022)
CUMS mice	5–20 mg/kg for 7 days	Decreasing the immobility time in the FST	Balancing neurotransmitters and decreasing the level of Glu	Guo-Li Wang et al. (2018a)
CUMS mice	4–10 mg/kg for 21 days	Decreasing immobility time in the FST and TST	Balancing neurotransmitters	Yamada et al. (2011)
CSDS mice	35–70 mg/kg daily orally for 28 days	Reversing the social avoidance behavior, anhedonia, and behavioral despair	Enhancing the BDNF signaling and upregulating hippocampal neurogenesis	Jiang et al. (2021a)
CRS rats	6.75–13.5 mg/kg	Ameliorating the memory impairments	Antioxidant; anti-apoptosis; improving synaptic plasticity; restoring the BDNF signaling	Jiang et al. (2021b)
CRS mice	10 mg/kg by intraperitoneal injection for 14 days	Relieving the depression-like behaviors	Anti-inflammation; pro-survival; increasing BDNF expression	Guo et al. (2021)
CMS-exposed mice	20 mg/kg for 4 weeks	Alleviating depressive-like behaviors	Inducing a pro-neurogenic phenotype of microglia, anti-inflammation	Zhang et al. (2021)

Ginsenoside Rb1 exerted promising antidepressant-like effects in mice with chronic social defeat stress (CSDS)-induced depression by enhancing the BDNF/Trkb signaling cascade, which increased the hippocampal neurogenesis (Jiang et al., 2021a). Similarly, ginsenoside Rb1 treatment ameliorated chronic restraint stress (CRS)-induced memory impairments in rats by improving synaptic plasticity and restoring the BDNF/TrkB signalling pathway (Jiang et al., 2021b). Guo et al. (2021) found that ginsenoside Rb1 not only increased the protein expression of BDNF, but also activated the pro-survival Akt pathway in CRS mice. In addition, ginsenoside Rb1 alleviated the inflammation induced by CRS, including decrease in the protein expression of IL-1β, TNF-α and ionized calcium binding adapter molecule 1 in hippocampus, and reduce in the levels of IL-1β and TNF-α in serum (Guo et al., 2021). Intervention with ginsenoside Rb1 for 2 weeks induced a pro-neurogenic phenotype of microglia *via* activating peroxisome proliferator-activated receptor γ (PPARγ), inhibited chronic mild stress (CMS)-induced inflammation and increased the proliferation and differentiation of neural precursor cells (Zhang et al., 2021) (Table 4).

These studies have demonstrated that ginsenoside Rb1 could improve the depressive-like behaviors through regulating the balance of neurotransmitters, inhibiting the neuroinflammation and promoting neuronal survival. Thus, ginsenoside Rb1 may be a promising candidate in the therapy of depression.

## Ginsenoside Rb1 in Spinal Cord Injury

Spinal cord, located in the spinal canal, is one part of the central nervous system. The spinal cord is comprised of white matter and gray matter, which includes cerebral nuclei and fiber conduction bundle. Spinal cord injury (SCI) is a central nervous system disease, which is classified as traumatic SCI (TSCI) and non-traumatic SCI (NTSCI), and this disease can lead to the permanent disability (Gedde et al., 2019). Currently, the prevalence of SCI witnesses a rise which makes a burden to the individual and the society because it is still incurable (Ahuja et al., 2017b). Since the functions of the spinal cord contain the motor adjustment and the sensory transmission, the position and the degree about SCI have different effect on the consequences. The causes of TSCI contain recreational activities, violence falls, traffic accident and so on (McDonald and Sadowsky, 2002) and that of NTSCI include compression of tumor, spinal canal stenosis, vascular ischemia, inflammatory conditions and so on (New and Sundararajan, 2008). Broadly speaking, the treatments of SCI contain neuroprotection and nerve regeneration (Kim et al., 2017). At present, the clinical treatment of SCI includes surgical decompression, drug therapy and early definitive care and so on (Ahuja et al., 2017a). Recently, more and more researches showed that ginsenoside Rb1 may have fine effects on the recovery of SCI.

Wang and others found that in SCI rats ginsenoside Rb1 treatment facilitated the expression of miR-130b-5p, which inactivated Toll-like receptor 4 (TLR4)/NF-κB in microglia and further increased Basso, Beattie, and Bresnahan score (BBBs) and reduced TUNEL-positive cell proportion and inflammation (Wang et al., 2021), suggesting that ginsenoside Rb1 alleviated SCI through reducing activated microglia-induced neuronal injury *via* miR-130b-5p/TLR4/NF-κB axis. Administration of ginsenoside Rb1 also significantly inhibited oxidative stress, including decreasing serum MDA content and increasing the activity of SOD, CAT and GSH, at least partly *via* the eNOS/Nrf2/HO-1 pathway in SCI rats, and improved spinal cord function score (Liu et al., 2018b). Ginsenoside Rb1 could improve neurological function of hind limbs and reduce the cell apoptosis through decreasing MDA content in serum and spinal cord tissue and increasing activity of SOD, as well as inhibit apoptosis by promoting the expression of Survivin protein in spinal cord I/R injury (SCII) rats (Ye et al., 2019). Importantly, Zhao et al., 2018a found that ginsenoside Rb1 injection after surgical operation also prevented neural cell apoptosis in the spinal cord and improved hindlimb locomotor dysfunction of SCII rats *via* inhibiting the activation of caspase-3 and apoptosis signal-regulating kinase (ASK 1), and the Bax/Bcl-2 ratio, suggesting that ginsenoside Rb1 may be a potential drug for SCII treatment. Sakanaka et al. (2007) produced dihydroginsenoside Rb1 and found that its effective dose to improve SCI was ten times lower than that of ginsenoside Rb1. Dihydroginsenoside Rb1 rescued SCI-induced neuronal damage through upregulating the expression of Bcl-x(L) and vascular endothelial growth factor (VEGF) by activating hypoxia response element (HRE) and signal transducers and activators of transcription 5 (Stat5), respectively. (Table 5).

**TABLE 5:** The neuroprotection of ginsenoside Rb1 in SCI.

Models	Dose of Rb1	Effects	Mechanisms	References
SCI rats	40 mg/kg at 30 min and 24 h after SCI treatment	Increasing BBBs and reducing TUNEL positive cell proportion	Inactivating microglia; anti-inflammation	Dan Wang et al. (2021a)
SCI rats	10 mg/kg at 30 min after modeling and then daily for 7 days	Improving spinal cord function score	Anti-oxidant; anti-inflammation	Xinwei Liu et al. (2018b)
SCII rats	10–80 mg/kg 30 min before SCII and the same do as before every day until being sacrificed	Improving neurological function of hind limbs	Anti-oxidant; anti-apoptosis; increasing survivin protein expression	Ye et al. (2019)
SCII rats	10 mg/kg after SCII model for 7 days	Improving hindlimb locomotor dysfunction of rats	Anti-apoptosis	Dongxu Zhao et al. (2018a)
SCI rats	1.2–6 μg/day of dihydroginsenoside Rb1 intravenously infused	Rescuing damaged neurons in spinal cord	Anti-apoptosis and upregulating VEGF expression	Sakanaka et al. (2007)
SCI rats	20 mg/kg for 2 weeks	Decreasing the loss of motor neurons, promoting function recovery	Inhibiting autophagy in neurons, anti-apoptosis	Peng Wang et al. (2018b)

Demyelination occurs after SCI due to the injury and the imbalance of the microenvironment while myelin promotes facilitate axon signal conduction. Study shows that the unceasing loss of oligodendrocytes may lead to the barrier to the function recovery, while the reason why the loss of oligodendrocytes happens owes to the imbalance between the remyelination and the demyelination (Fan et al., 2018). Ginsenoside compound K, a metabolite of ginsenoside Rb1, promoted the proliferation, migration and differentiation of Schwann cells, which are critical for the remyelination of injured peripheral nerve, *via* activating MEK/ERK1/2 and PI3K/Akt pathways (Wang et al., 2021). Autophagy is the process of engulfing unwanted cells and maintaining homeostasis. A study shows that autophagy may lead to the death of the oligodendrocytes while functional recovery requires the participation of oligodendrocytes (Fan et al., 2018). The maintenance of neurons requires the participation of autophagy. However, high levels of autophagy may lead to neuronal cell death and further cause neurodegeneration. Bisicchia and his others demonstrated that inhibition of autophagy improved the survival rate of rats with spinal cord hemisection (Bisicchia et al., 2017). Wang et al. (2018b) found ginsenoside Rb1 treatment decreased the death of motor neurons and promoted the recovery of motor function, as well as restored the expression of LC3-II/I, Beclin-1, and p62 in the SCI rats, suggesting that ginsenoside Rb1 may play neuroprotective role *via* inhibiting autophagy and autophagic cell death. (Table 5). These data suggest that ginsenoside Rb1 may be a promising candidate in the therapy of SCI.

## Conclusion and Expectation

**FIGURE 2:**
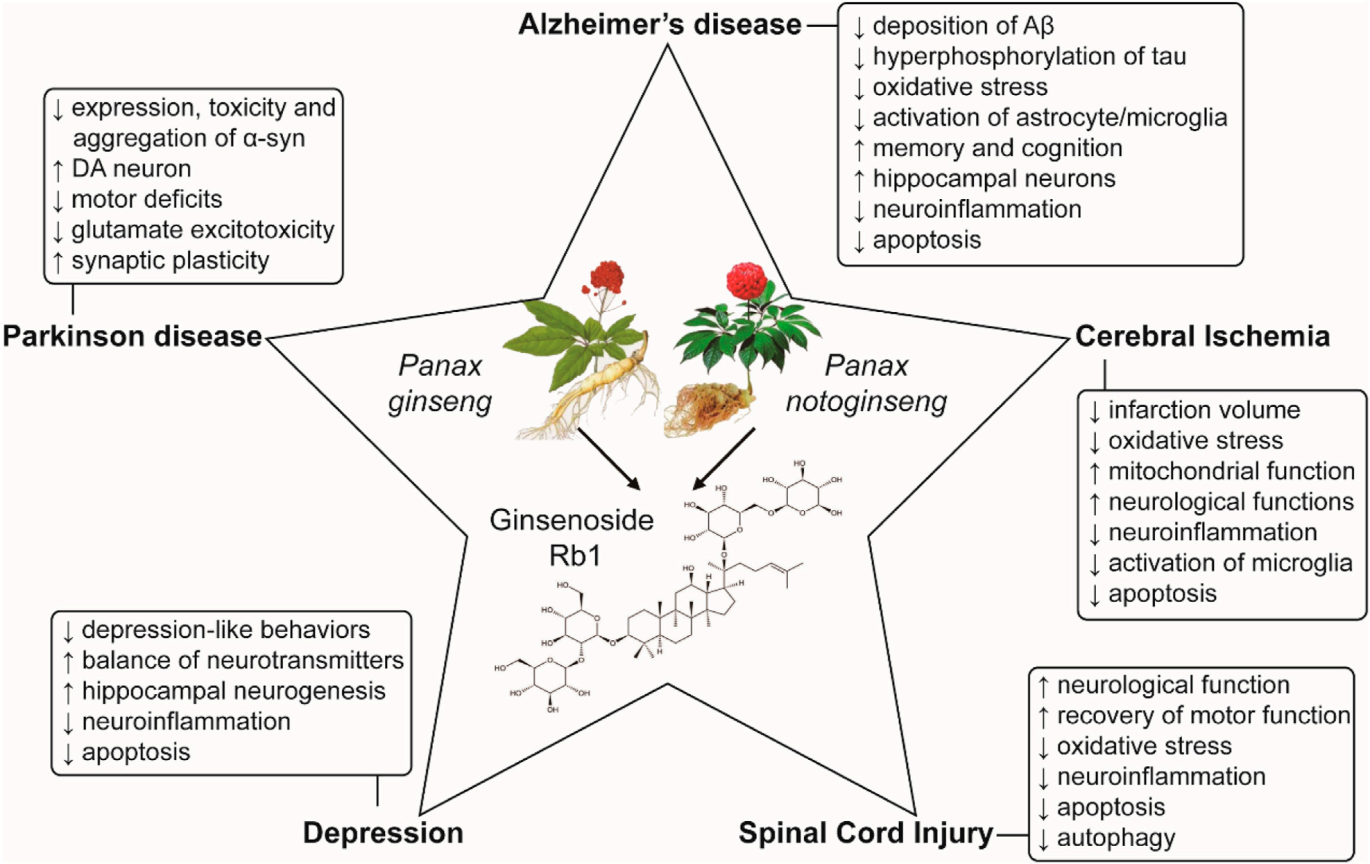
Protective mechanisms of ginsenoside Rb1 in CNSDs.

In summary, current studies have demonstrated that ginsenoside Rb1 exerts neuroprotective roles through inhibiting oxidative stress, apoptosis and neuroinflammation and regulating the autophagy in neurodegenerative diseases, cerebral ischemia injury, depression and spinal cord injury (Figure 2). More importantly, the effects of ginsenoside Rb1 seems to be much stronger than other ginsenosides, suggesting that may be a promising candidate agent for clinical therapy of CNSDs.

At this stage, the associated studies are extremely few and limited to focus on the neuroprotective effects of ginsenoside Rb1 in CNSDs in cellular and rodent models. The experiments in nonhuman primate models of CNSDs and the clinical trials should be also successively carried out to accelerate the clinical application of ginsenoside Rb1 for CNSDs treatment in the future. There are different routes of administration and the dosage used in rodents is in a large range. Further studies are also needed to performed to confirm the optimal administration route and dosage for individual disease.
